# Physical activity and depressive symptoms after stillbirth: informing future interventions

**DOI:** 10.1186/s12884-014-0391-1

**Published:** 2014-11-29

**Authors:** Jennifer Huberty, Jenn A Leiferman, Katherine J Gold, Lacey Rowedder, Joanne Cacciatore, Darya Bonds McClain

**Affiliations:** Exercise and Wellness, Arizona State University, Phoenix, AZ USA; Community and Behavioral Health, Colorado School of Public Health, Aurora, CO USA; Department of Family Medicine, University of Michigan, Ann Arbor, MI USA; Department of Obstetrics & Gynecology, University of Michigan, Ann Arbor, MI USA; School of Social Work, Arizona State University, Phoenix, AZ USA; College of Nursing and Health Innovation, Arizona State University, Phoenix, AZ USA

**Keywords:** Stillbirth, Perinatal loss, Exercise, Yoga, Mental health

## Abstract

**Background:**

In the United States, approximately one in 110 pregnancies end in stillbirth affecting more than 26,000 women annually. Women experiencing stillbirth have a threefold greater risk of developing depressive symptoms compared to women experiencing live birth. Depression contributes negatively to health outcomes for both mothers and babies subsequent to stillbirth. Physical activity may improve depression in these women, however, little is known about acceptable physical activity interventions for women after stillbirth. This is the purpose of this descriptive exploratory study.

**Methods:**

Eligible women were between ages 19 and 45, and experienced stillbirth within one year of the study. An online survey was used to ask questions related to 1) pregnancy and family information (i.e., time since stillbirth, weight gain during pregnancy, number of other children) 2) physical activity participation, 3) depressive symptomatology, and 4) demographics.

**Results:**

One hundred seventy-five women participated in the study (*M* age = 31.26 ± 5.52). Women reported participating in regular physical activity (at least 150 minutes of moderate activity weekly) before (60%) and during (47%) their pregnancy, as well as after their stillbirth (61%). Only 37% were currently meeting physical activity recommendations. Approximately 88% reported depression (i.e., score of >10 on depression scale). When asked how women cope with depression, anxiety, or grief, 38% said physical activity. Of those that reported using physical activity to cope after stillbirth, they did so to help with depression (58%), weight loss (55%), and better overall physical health (52%). To cope with stillbirth, women used walking (67%), followed by jogging (35%), and yoga (23%). Women who participated in physical activity after stillbirth reported significantly lower depressive symptoms (*M* = 15.10, *SD* = 5.32) compared to women who did not participate in physical activity (*M* = 18.06, *SD* = 5.57; *t* = -3.45, *p* = .001).

**Conclusions:**

Physical activity may serve as a unique opportunity to help women cope with the multiple mental sequelae after stillbirth. This study provides data to inform healthcare providers about the potential role of physical activity in bereavement and recovery for women who have experienced stillbirth. Additional research is necessary in this vulnerable population.

## Background

Approximately one in 110 pregnancies end in stillbirth (intrauterine death of a baby at or after the 20^th^ completed gestational week), affecting more than 26,000 women annually [1,2] in the United States. After giving birth (both live and stillbirth), women may experience significant physical, mental, and social maladies including alterations in appetite and sleep patterns, guilt and shame, and depression [3-5]. However, women who experience stillbirth are at three times the risk of developing depressive symptoms compared to women who had a live birth [5-7]. In a recent study, 7-8% of women with a live birth experienced symptoms of posttraumatic stress compared to 44% of women who experienced stillbirth [8]. Depressive states may contribute to excessive weight retention/gain, chronic disease risk (i.e., heart disease, diabetes), premature mortality, and can negatively impact the health of babies born subsequent to stillbirth [9]; the majority of which (50-98%) are conceived within a year after stillbirth [10-12]. Women who have experienced stillbirth are medically high-risk and vulnerable, underscoring the need to identify efforts to enhance their mental and physical health.

Inter-conception care is provided to women of reproductive age between pregnancies. The goal of inter-conception care is the same as pre-conception care, to ensure that conditions and behaviors, which may pose maternal and infant risks, are identified and managed [13]. Unfortunately, inter-conception interventions to improve the mental and physical health of women who have experienced a stillbirth are essentially non-existent. Most often treatment for bereaved mothers is mirrored after women who have experienced a live birth (i.e., return for a 6-week check-up) and may include psychiatric medications [14], and/or, when available, referral to social support groups [15]. Thus, the development of inter-conception interventions specifically designed to decrease negative psychiatric sequalae and increase positive coping after stillbirth is critical to individual, familial, and public health.

Physical activity improves depressive symptoms in a number of populations and has been shown to be more efficacious than psychiatric medications [16-18]. Additionally, women who are active during and after pregnancy have fewer depressive symptoms and report better mood as compared to inactive pregnant and post-partum women [19-21]. It is unknown whether this also applies to women who have experienced stillbirth. In a study by Huberty et al. [22], women who have experienced a stillbirth who reported regular physical activity described mental, emotional, and physical benefits that helped them better cope with grief [22]. The same mothers also felt that activity helped them better cope with emotional stressors related to stillbirth [23]. Even mothers who were not regularly active reported that when they were active they felt better, had a better mindset, and had more energy [22]. The literature suggests a number of mechanisms for the physical activity-depression relationship including elevated body temperature, increases in endorphins and neurotransmitters, and improvements in self-efficacy [23]. Some of these same mechanisms may occur in women who have experienced stillbirth.

Unfortunately, little is known about what type of physical activity interventions are acceptable for women who experienced stillbirth. Women with a live birth report lack of time, motivation, child-care, and other adjustments to parenting (i.e., more responsibility, prioritizing) as major barriers to physical activity participation [24-27]. However, women who experienced stillbirth report barriers to physical activity participation unique to stillbirth [22] such as emotional symptoms (i.e., feeling depressed, emotionally drained) and diminished motivation, being tired, feeling guilt, having a pregnant body with no baby, and being confronted with other babies (i.e., exercise in public settings, outside the home) [22]. Understanding the specific physical activity preferences for these women could inform targeted physical activity interventions.

Therefore, the purpose of this study was to learn more about women’s desire to use physical activity to improve depressive symptoms after stillbirth, and their experience with physical activity after stillbirth. Findings from this study will be used to inform the design of inter-conception interventions to improve mental and physical health in these women.

## Methods

This was a descriptive exploratory study that used a convenient non-probabilistic sample. The Institutional Review Board at Arizona State University approved the study.

### Participants

Women were eligible to participate in the study if they were 19 to 45 years of age, lived in the United States, spoke English, and experienced the death of a baby to stillbirth within one year of participation. Current pregnancy was not an exclusion from the study. Women were recruited locally and nationally through collaboration with non-profit stillbirth advocacy organizations (e.g., Star Legacy Foundation, Still Standing Magazine), posting flyers at venues (e.g., hospitals, physician clinics) and snowball sampling (i.e., sharing information about the studies with others) through social media (e.g., Facebook, Twitter).

### Procedures

Recruitment for the study occurred between February 2014 and July of 2014. The survey was free, voluntary, and available online. Women who chose to complete the survey either entered a link online from the recruitment materials (i.e., fliers) or clicked on an internet link (e.g., Facebook, email). The survey was hosted by Qualtrics (Provo, Utah). Completion of the survey was taken as consent. Participants were allowed to choose no response or skip questions in the survey. At the end of the survey, participants had the opportunity to provide their email address if they wished to be contacted for future studies.

### Survey

The survey included the following four sections: (1) Pregnancy and family information (n = 5 items). Questions in this section were related to time since stillbirth, weight gain during pregnancy, and number of other children. (2) Physical activity participation over time (i.e., before pregnancy, during pregnancy, after stillbirth; n = 6 items). An example question includes, “In the time since your loss, have you participated in exercise or physical activity?” (3) Physical Activity and Depressive Symptomatology (n = 20 items). Questions were related to a history of diagnosis of depressive mood disorder and coping with depressive symptoms (i.e., preferences for physical activity). (4) Demographics (i.e., race, ethnicity, income, education; n = 8 items).

To define and assess physical activity before, during, and after their most recent pregnancy as well as currently, a modified version of the Physical Activity Stages of Change Questionnaire was used [28]. The Edinburgh Postnatal Depression Scale (EPDS) was used to determine depressive symptomatology and is a reliable and valid tool for use in women who experienced stillbirth [29]. Scores above 13 on the EPDS indicated high depressive symptomatology [30].

### Data analysis

Descriptive statistics (e.g., means, percentages) for all the survey questions were calculated. The number of women currently meeting physical activity recommendations of 150 minutes of moderate intensity physical activity [31] each week was determined by multiplying the amount of days they reported being active by the time per day spent practicing physical activity. Options for the “time per day” question were expressed in a range (i.e. 15-29 minutes), and the lowest number in the range was used for the calculation. An independent t-test was used to compare average level of self-reported depressive symptoms in women who participated in physical activity and was compared to those that did not participate in physical activity since experiencing stillbirth. A linear regression model estimated the relationship between physical activity after stillbirth while controlling for time since stillbirth and history of depressive disorder diagnosis. A chi-square test was conducted to compare the proportion of women who scored above the depression threshold on the EPDS across physical activity groups (active (meeting recommendations of 150 minutes per week) [31] as compared to inactive (i.e., not meeting recommendations) since stillbirth).

## Results

### Participant characteristics

Three hundred fifty two women clicked on the link to participate in the survey. Of these 297 completed the survey (84%), 38 were excluded for residency outside of the U.S., six were excluded for being over the age of 45 years, 49 were excluded for being more than one year since stillbirth, and 29 had missing data related to the date of their stillbirth. A total of 175 (50%) of women who had experienced stillbirth in the past year were included in the final analysis and were between the ages of 19-45 years (mean 31.26 ± 5.52 years). A majority of the women were married (83%), Caucasian (86.5%) and were living in the southern portion of the United States (43%). One-third of our sample reported prior diagnosis with a depressive mood disorder and of those 59% had been diagnosed within the past year. Almost 40% of our sample had experienced their stillbirth within the last three months. All demographics are presented in Table 1.

**Table 1:** **Demographics of participants (N = 175)**

	**n %**
Zip code (n = 120)	
West	27 (22.5)
Midwest	30 (25.0)
Northeast	11 (9.2)
South	52 (43.3)
Race (n = 155)	
Caucasian	134 (86.5)
African American	5 (3.2)
Asian	1 (0.6)
American Indian	3 (2.0)
Other	12 (7.7)
Education level (n = 155)	
<High School	3 (2.0)
High School Diploma	23 (14.8)
Some College	35 (22.6)
Associates Degree	25 (16.1)
Bachelors Degree	33 (21.3)
Graduate School+	36 (23.2)
Income (n = 154)	
<$20,000	26 (16.9)
$21,000-$40,000	44 (28.6)
$41,000-$60,000	23 (14.9)
$61,000+	61 (39.6)
Marital Status (n = 155)	
Single	6 (3.9)
Partnered/In a relationship	20 (12.9)
Married	128 (82.6)
Divorced	1 (0.6)
Age (n = 169)	mean = 31.62 ± 5.52
18-24	26 (15.4)
25-29	40 (23.7)
30-34	58 (34.3)
35-39	34 (20.1)
40-45	11 (6.5)
Time since loss (n = 175)	
0-3 months	68 (38.9)
4-6 months	34 (19.4)
7-9 months	41 (23.4)
10-12 months	32 (18.3)

### Physical activity participation and depressive symptoms

Information about using physical activity to cope with depressive symptoms is presented in Table 2. Approximately 60% of the sample reported participating in regular physical activity (meeting recommendations of 150 minutes per week) [31] before their pregnancy and 47% participated during their pregnancy. Of the women reporting participation in physical activity since their stillbirth (n = 104, 61%), only 38 women (37%) met current physical activity recommendations of at least 150 minutes weekly. Most women reported engaging with friends/family (72%), participating in group support (52%), and physical activity (38%) as a means to cope with their depressive symptoms. Of those that reported engaging in physical activity, the most important reasons were to help with depressive symptoms (58%), lose weight (55%), and to have better overall physical health (i.e., fitness) (52%). When asked what type of physical activity women used to cope with their depressive symptoms, most said walking (67%), followed by jogging (35%), and yoga (23%). Although less than 20% of women reported trying yoga as a means to cope with depressive symptoms, almost 50% were interested in using yoga to cope and of these, 80% would prefer to do yoga in their home.

**Table 2:** **Physical activity and depression (N = 175)**

		**n %**
Participated in PA BEFORE pregnancy (n = 171)	Yes	102 (59.6)
	No	69 (40.4)
Participated in DURING pregnancy (n = 171)	Yes	80 (46.8)
	No	91 (53.2)
Participated in PA since loss (n = 171)	Yes	104 (60.8)
	No	67 (39.2)
Meeting physical activity recommendations (n = 104)	Yes	38 (36.9)
	No	65 (63.1)
Ever diagnosed with a depressive mood disorder? (n = 167)	Yes	56 (33.5)
	No	111 (66.5)
Mean depression score by time since loss (n = 167)		
0-3 months (n = 64)		17.58 ± 5.22
4-6 months (n = 33)		14.67 ± 5.81
7-12 months (n = 40)		15.73 ± 5.30
0-12 months (n = 30)		15.97 ± 6.15
How do you currently cope with depressive symptoms, anxiety, and/ or grief associated with the loss of your baby? (n = 175)		
Engaging/talking with family/friends		126 (72.0)
Support group		92 (52.5)
Physical activity (i.e., running, walking, yoga)		66 (37.7)
Therapist/counselor		64 (36.5)
Other (please specify)		40 (22.8)
Anti-depressive medication		39 (22.3)
Meditation		20 (11.4)
What type of physical activity specifically do you use to cope? (Check all that apply) {Responses only included if they answered “physical activity” to the question above} (n = 66)		
Brisk walking		44 (66.7)
Jogging or running		23 (34.8)
Other (please specify)		15 (22.7)
Yoga		15 (22.7)
Hiking		11 (16.7)
Biking		7 (10.6)
Aerobics or aerobic dancing		6 (10.9)
Swimming		6 (9.1)
Reasons for participating in physical activity ranked from 1 (most important) to 4 (least important) (Ranks 1 and 2 are combined and reported here) (n = 64)		
Help with depressive symptoms, anxiety, grief		37 (57.8)
Weight loss		35 (54.7)
Better overall physical health (i.e., fitness)		33 (51.6)
Better quality of life		26 (40.6)
Other (please specify)		6 (93.8)
Have you ever tried to use yoga as a means to “cope” with depressive symptoms, anxiety, and/or grief associated with the loss of your baby? (n = 166)		
	Yes	29 (17.5)
	No	137 (82.5)
Are you interested in using yoga to cope with depressive symptoms, anxiety, and/or grief associated with the loss of your baby? (n = 137)		
	Yes	66 (48.2)
	No	71 (51.8)
Rank the settings in which you would prefer to participate in yoga from 1 to 4. (Ranks 1 and 2 are combined and reported here) (n = 66)		
In your own home		53 (80.3)
Yoga studio		36 (54.5)
Group-based setting at hospital/doctors office		17 (25.8)
Gym		17 (25.8)
Other (please specify)		3 (5.0)

### Relationship between physical activity participation and depression scores

Women who reported participating in regular physical activity since their stillbirth reported significantly lower levels of depressive symptoms (Mean (*M*) = 15.10, *SD* = 5.32) compared to women who did not participate in regular physical activity (*M* = 18.06, *SD* = 5.57; *t* = -3.45, *p* = .001). Linear regression model results (F =10.79, p = .00; *R*^2^ = .17) indicated that the relationship between physical activity and depressive symptoms remained significant (*β* = -.26, *p* = .00) after controlling for time since stillbirth (*β* = -.12, *p* = .09) and history of depressive disorder diagnosis (*β* = .30, *p* = .00). There was also a significant difference when comparing the proportion of women scoring above the depression threshold who participated physical activity since the stillbirth (57%) versus those who did not participate in physical activity (78%; χ^2^ = 7.43, *p* = .01).

## Discussion

This was the first study to determine women’s preferences for and experiences with physical activity after stillbirth. As such, it offers information necessary for healthcare providers to design and implement inter-conception interventions that include physical activity to improve the mental and physical health of women after stillbirth, an area which is lacking. Women who have experienced stillbirth are a high-risk population for poor maternal and infant health outcomes because of the negative health implications (i.e., depressive symptoms, weight retention) due to the traumatic event (i.e., stillbirth) and the high rate of conceiving within a year after the stillbirth. The encouragement of positive health behaviors such as physical activity in women who have experienced stillbirth during inter-conception care may positively impact maternal and fetal health outcomes in subsequent pregnancies. Unfortunately, health care providers are not prescribing physical activity for women who have experienced stillbirth. Women who have experienced stillbirth have reported that physicians do not counsel about exercise beyond “returning to normal activity” and that they would have liked information about physical activity and/or advice from their physician about being active for their health after their stillbirth [22]. The promotion of positive health behaviors and the positive implications of this is evident in the literature related to women of live births that are active before, during, and after their pregnancies [32,33]. It is important to understand the specific and unique needs of women who have experienced stillbirth in order to design interventions to help improve their mental, and physical health outcomes.

Despite a decrease in the number of women who reported participating in regular physical activity during their pregnancy (47%) compared to before their pregnancy (60%), 61% of women reported participating in regular physical activity since their stillbirth. This may have been because women who were active knew that exercise might help them feel better [22]. In a study by Huberty and colleagues [22], women who experienced stillbirth and were active prior to their stillbirth reported using physical activity to cope, feel better, and have time alone [22]. This may also be because despite needing to physically recuperate, low to moderate intensity physical activity (i.e., walking, yoga) offers an outlet in which to feel better and have alone time [22]. This is important, as exercise is a well-known non-pharmacological method to decrease depressive symptoms and reduce episodic recurrences [16,34]. However, in our sample less than half of women who reported being active since stillbirth were currently meeting recommendations for physical activity. This is similar to other studies in non-bereaved women during pregnancy and postpartum [35]. It is well known that pregnant women are less active than non-pregnant women and that pregnancy leads to a decrease in physical activity participation [26]. Additionally, physical activity levels post-partum may not return to what they were pre-pregnancy [36,37]. Strategies are needed to help encourage and guide women who experienced stillbirth to use physical activity as a means to cope and to help them maintain this activity in the long-term. This is especially important because our study suggests that women who have experienced stillbirth and participate in regular physical activity have less depressive symptoms than women who are not regularly active. Similar findings were reported in women who have postpartum depression after a live birth and participate in exercise programs [19,38]. Thus, healthcare providers may consider suggesting exercise after a mother experiences stillbirth as a way to cope with grief and depressive symptoms. Additional research in this area is warranted.

In our study, 38% of women reported using activity as a means to cope with depressive symptoms, anxiety, and/or grief associated with the death of their baby. Others reported engaging with family/friends, and support groups to cope with their symptoms. Medical and mental health providers may consider offering group-based exercise programs for women who have experienced stillbirth that incorporate both a social support and exercise component. For example, support groups’ session times could be shortened (30 minutes as opposed to 60 minutes) and 30 minutes of yoga or walking could be incorporated. Similar approaches were used in chronic conditions and social support (i.e., group-based) was a known facilitator to improving physical activity participation [39-41]. More research is warranted.

Yoga has burgeoned as an exercise trend in Western society because it positively affects psychological conditions, pain, cardiovascular, autoimmune and immune conditions, and pregnancy outcomes. Based on findings in studies in pregnant and postpartum women and other chronic conditions, yoga represents a potential means of care that may help women build their capacity to be resilient and cope with depressive symptoms both immediately after their stillbirth and in the months and years that follow [41-43]. Even though less than 20% of women in this study used yoga to cope, 50% were interested in yoga and of those, most preferred yoga in their home. Studies that examine the feasibility of in-home versus in-studio yoga settings may further inform the design of interventions and help guide low-cost, sustainable strategies for coping after stillbirth.

Our study was the first to describe women’s preferences for using physical activity to cope with depressive symptoms after stillbirth. Strategies for interventions to improve the mental and physical health in these women are lacking and the information from this study fills a gap in our knowledge in this area. However, there are some limitations to our study. First, our sample was predominantly Caucasian, educated, and married, therefore the generalizability of our findings to underserved and non-married populations are limited. Second, because the survey was internet-based our sample was limited to those who had access to the Internet and may have been seeking out or already using pregnancy loss support resources. Third, our sample had higher rates of reported physical activity prior to their pregnancy as compared to national levels of physical activity in women of reproductive age [19-21]. This may be because women who were already participating in physical activity or more interested in it may have been more likely to complete the survey. Additionally, physical activity was self-reported and it is well known that self-reported physical activity is often overestimated compared to objectively measured physical activity [44-46]. Given that this study was cross-sectional, we cannot infer the direction of effects from our linear regression model results. Finally, women who experienced stillbirth more than one year ago were not included in this study and data suggests that these women have levels of depressive symptoms comparable to women within the first year [15]. Information from women post one year from stillbirth in relation to preferences for physical activity could also be very useful to inform future interventions.

## Conclusion

Women who have experienced stillbirth experience negative mental and physical health outcomes as a result of their stillbirth, many of which may impact subsequent pregnancies. Current inter-conception interventions are non-existent or based upon women who have experienced live births. Women who have experienced stillbirth may be interested in physical activity interventions to help them cope, feel better, and have alone time. Additionally, women may be interested in improving their mental (i.e., depressive symptoms) and physical (i.e., weight) health for subsequent pregnancies. Women report using activity to cope and are most interested in walking, jogging, or yoga interventions, all of which are low cost, non-pharmacological solutions to help improve depressive symptoms. Physical activity interventions may provide opportunities for health care professionals to help women cope with depressive symptoms after their stillbirth. More research in this area is warranted.
